# Impact of Preexisting Adenovirus Vector Immunity on Immunogenicity and Protection Conferred with an Adenovirus-Based H5N1 Influenza Vaccine

**DOI:** 10.1371/journal.pone.0033428

**Published:** 2012-03-14

**Authors:** Aseem Pandey, Neetu Singh, Sai V. Vemula, Laurent Couëtil, Jacqueline M. Katz, Ruben Donis, Suryaprakash Sambhara, Suresh K. Mittal

**Affiliations:** 1 Department of Comparative Pathobiology, Purdue University, West Lafayette, Indiana, United States of America; 2 Department of Clinical Veterinary Sciences, College of Veterinary Medicine, Purdue University, West Lafayette, Indiana, United States of America; 3 Bindley Bioscience Center, Purdue University, West Lafayette, Indiana, United States of America; 4 Influenza Division, Centers for Disease Control and Prevention, Atlanta, Georgia, United States of America; Virginia Polytechnic Institute and State University, United States of America

## Abstract

The prevalence of preexisting immunity to adenoviruses in the majority of the human population might adversely impact the development of adaptive immune responses against adenovirus vector-based vaccines. To address this issue, we primed BALB/c mice either intranasally (i.n.) or intramuscularly (i.m.) with varying doses of wild type (WT) human adenovirus subtype 5 (HAd5). Following the development of immunity against HAd5, we immunized animals via the i.n. or i.m. route of inoculation with a HAd vector (HAd-HA-NP) expressing the hemagglutinin (HA) and nucleoprotein (NP) of A/Vietnam/1203/04 (H5N1) influenza virus. The immunogenicity and protection results suggest that low levels of vector immunity (<520 virus-neutralization titer) induced by priming mice with up to 10^7^ plaque forming units (p.f.u.) of HAd-WT did not adversely impact the protective efficacy of the vaccine. Furthermore, high levels of vector immunity (approximately 1500 virus-neutralization titer) induced by priming mice with 10^8^ p.f.u. of HAd-WT were overcome by either increasing the vaccine dose or using alternate routes of vaccination. A further increase in the priming dose to 10^9^ p.f.u. allowed only partial protection. These results suggest possible strategies to overcome the variable levels of human immunity against adenoviruses, leading to better utilization of HAd vector-based vaccines.

## Introduction

Adenoviruses (Ad) possess several attributes that make them suitable candidates for vaccine vectors [1], [2]. Ad exert an adjuvant-like effect by stimulating the innate immune system through both Toll-like receptor (TLR)-dependent and TLR-independent pathways [3], [4]. The effectiveness of Ad vector-based vaccines against many infectious diseases, including measles, severe acute respiratory syndrome (SARS), human immunodeficiency virus (HIV), hepatitis B and Ebola has been evaluated in animal models and clinical trials in humans [5]–[9]. Previously, we and others have explored the potential of a human Ad serotype 5 (HAd5) vector-based vaccine strategy for H5N1 influenza [10]–[12]. Our immunogenicity and protective efficacy studies demonstrated that Ad vector-based vaccines provide complete protection against challenge with homologous and antigenically distinct strains of influenza viruses in a mouse model [11].

There is a high incidence of Ad infections in the general population due to the circulation of more than fifty Ad serotypes. Their ubiquitous nature results in the development of Ad-specific neutralizing antibodies, popularly known as ‘preexisting vector immunity’ in the majority of the individuals [13]–[15]. Ad-neutralizing antibodies inhibit the vector extracellularly, while Ad-specific CD8+ T cells destroy vector expressing cells [16], [17] thereby adversely impacting the duration and levels of transgene expression. Experimental studies in animal models have shown that in the presence of extremely high levels of Ad-neutralizing antibodies, there is a significant inhibition in the development of immunogen-specific immune responses [18]. A comprehensive analysis of Ad seroprevalence found that HAd5 neutralizing antibody titers in the study's participants varied by geographic location and ranged from 18 to 4690 [19]. According to this study, 26% of the participants had titers below 200, 40% had titers below 1000, and 20% exhibited titers greater than 1000. These studies have underscored the need to further evaluate the role of vector immunity in inhibiting the immunogenicity and efficacy of HAd vector-based vaccines.

To determine the level of vector immunity that can be tolerated without significantly affecting the vaccine efficacy, we primed groups of mice with varying doses of wild type (WT) HAd5 via intranasal (i.n.) or intramuscular (i.m.) route of inoculation to generate different levels of HAd5-neutralizing antibody titers. After the development of HAd5-specific immunity, HAd-primed mice were immunized i.n. or i.m. with a low or high dose of a HAd vector (HAd-HA-NP) carrying the hemagglutinin (HA) and nucleoprotein (NP) genes of the A/Vietnam/1203/04 (H5N1) influenza virus. We also assessed if we could overcome vector immunity by increasing the vaccine dose and changing the route of immunization. Our results suggest that a high level (up to a neutralization titer of 2240) of vector immunity can be tolerated or effectively overcome by increasing the vaccine dose or using alternate routes of vaccination.

## Results

### Generation and characterization of HAd vector expressing HA and NP of H5N1 influenza virus (HAd-HA-NP)

**Figure 1:**
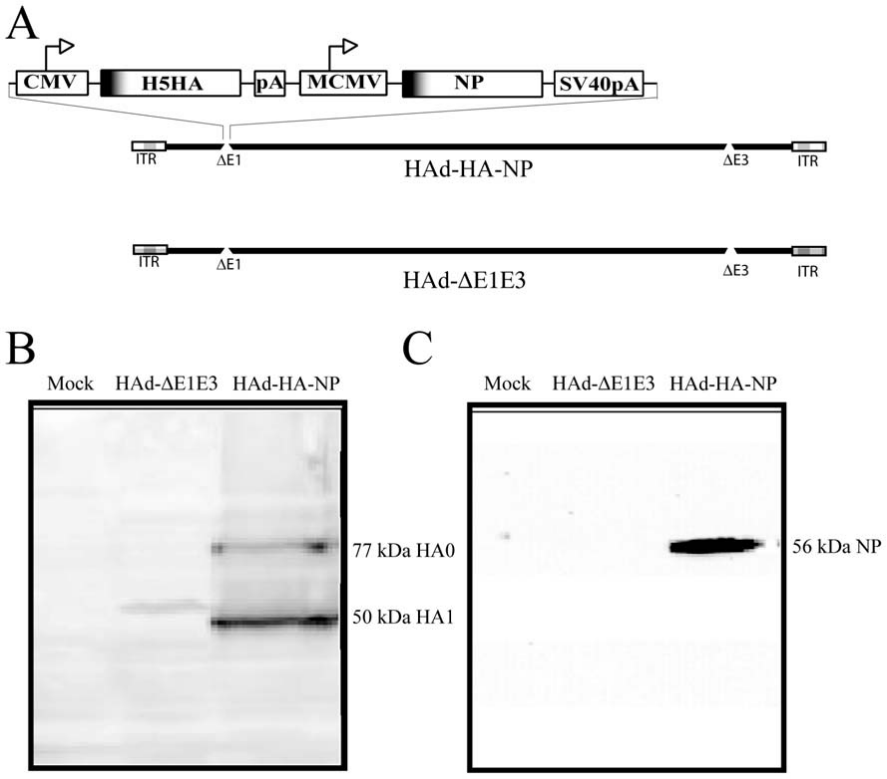
Replication-defective HAd vector (HAd-HA-NP) expresses HA and NP of a H5N1 influenza virus in vector-infected cells. (A) Diagrammatic representation of replication-deficient HAd vectors, HAd-ΔE1E3 [HAd5 with deleted E1 and E3 regions] and HAd-HA-NP [HAd-ΔE1E3 with hemaggluttinin (HA) and nucleoprotein (NP) gene from A/Vietnam/1203/04 (H5N1) influenza virus]. ITR, inverted terminal repeat; CMV, cytomegalovirus immediate early promoter; pA, polyadenylation signal; MCMV, mouse cytomegalovirus immediate early promoter; SV40pA, simian virus polyadenylation signal. (B and C) Expression of H5N1 HA and NP in 293 cells infected with HAd-HA-NP. Mock (PBS-infected), HAd-ΔE1E3-, or HAd-HA-NP-infected 293 cells were harvested 48 h post-infection, and cell lysates were analyzed by Western blot using polyclonal serum against H5 HA or a NP-specific mouse monoclonal antibody.

The full coding region of HA under the control of the cytomegalovirus (CMV) immediate early promoter and bovine growth hormone (BGH) polyadenylation signal (polyA) and full length coding region of NP gene of the A/Vietnam/1203/04 virus under the control of the murine CMV promoter and the simian virus 40 (SV40) polyA were inserted into early region 1 (E1) of the HAd genome using the Cre-recombinase-mediated site-specific recombination system [20]. Both genes in HAd-HA-NP were in the E1-parallel orientation. The recombinant vector, HAd-HA-NP (Figure 1A) showed visible cytopathic effect (c.p.e.) on the ninth day post-transfection. Western blot analysis was done to confirm the expression of HA and NP in 293 cells. Two distinct polypeptide bands of approximate molecular weights 77 kDa and 50 kDa, representing the HA precursor (HA0) and a proteolytic cleavage product (HA1), respectively, (Figure 1B) were observed in the HAd-HA-NP infected 293 cell lysate. A single band at approximate molecular weight of 56 kDa representing NP (Figure 1C) was visible in the HAd-HA-NP infected 293 cell lysate.

### Generation of HAd-primed mice having HAd neutralizing antibodies

**Figure 2:**
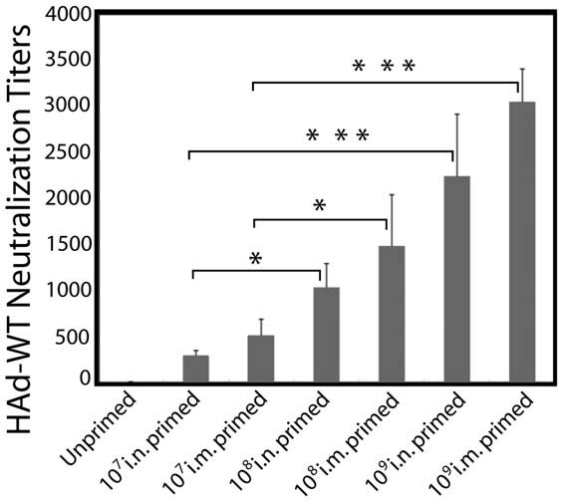
Development of vector immunity in wild type (WT) HAd5-primed animals. To induce HAd vector–specific immunity in mice, 6–8 weeks old female BALB/c mice were primed intramuscularly (i.m.) or intranasally (i.n.) with a single dose of 10^7^, 10^8^, or 10^9^ p.f.u. of HAd-WT. The unprimed mice received PBS. Four weeks after priming, mice were bled by retro-orbital puncture to evaluate the development of HAd-specific neutralizing antibodies by virus neutralization assay. Virus neutralization titers were the reciprocal of the highest serum dilution that completely prevented the development of c.p.e. The error bars represent Mean ± SD from five animals/group: *, *P*≤0.05; ***, *P*≤0.005.

To mimic in a mouse model the preexisting immunity against HAd5 observed in the majority of the human population, groups of animals were inoculated i.n. or i.m. with a single dose of 10^7^, 10^8^, or 10^9^ plaque forming units (p.f.u.) HAd-WT. Both i.n. and i.m. primed groups showed a dose-dependent increase in the levels of HAd-specific neutralizing antibody titers (Figure 2). As expected, the highest HAd-specific neutralizing antibody titers in i.n. inoculated HAd-primed groups were observed with a 10^9^ p.f.u. dose of HAd-WT (2240) followed by 10^8^ p.f.u. (1040) and 10^7^ p.f.u. (300) dose (Figure 2). Similarly, the i.m. primed group receiving 10^9^ p.f.u. of HAd-WT developed the highest titer (3040) followed by 10^8^ p.f.u. (1480) and 10^7^ p.f.u. (520) dose groups (Fig. 2). The i.m. primed groups resulted in the development of higher levels of HAd-specific neutralizing antibody titers compared to the i.n. primed groups.

### Induction of humoral immune response in HAd-primed mice immunized with HAd-HA-NP

Development of a robust HA-specific antibody response is an important indicator of the immunogenicity and protective efficacy of an influenza vaccine [21]. The i.n. or i.m. immunization of naïve animals with HAd-HA-NP elicited dose-dependent geometric mean (GM) hemagglutination inhibition (HI) titers (Table 1). The 10^7^ i.n. HAd-primed groups developed significantly high serum HI titers compared to the vector control group by the i.n. (120) or i.m. (160) routes of immunization with 10^8^ p.f.u. of HAd-HA-NP. Similarly, 10^7^ i.m. HAd- primed groups developed significantly higher (*P*≤0.05) serum HI titers compared to the vector control group by both i.m. (105) and i.n. (91) routes of immunization with HAd-HA-NP. The serum HI titers in 10^7^ HAd-primed groups (i.n. or i.m.) immunized with the vaccine were 1.5–2 fold lower than the naïve immunized groups indicating that HAd-preexisting antibodies affected the induction of humoral immune response following immunization with HAd-HA-NP. In the 10^8^ i.n. HAd-primed group, i.n. immunization with HAd-HA-NP induced lower serum HI titers (22) compared to the group immunized i.m. with HAd-HA-NP (80). Interestingly, an increase in the vaccine dose by five-fold resulted in significant (*P*≤0.05) enhancement of serum HI titers in mice immunized either i.n. (91) or i.m. (105). A similar trend was observed in the case of 10^8^ i.m. HAd-primed groups indicating that alternating the route of priming and immunization or increasing the vaccine dose can partially overcome the vector immunity.

**Table 1:** Hemagglutination inhibition (HI) antibody titers before challenge and lung viral titers 3 days post-challenge with a homologous H5N1 reassortant virus in HAd-HA-NP-immunized naïve and HAd-primed mice.

Preimmunization	Immunization	HI titers (GM)	Log_10_EID_50_/ml ± S.D.
10^9^ HAd-WT (i.n.)	5×10^8^ HAd-ΔE1E3 (i.n.)	10	6.5±0.1
10^7^ HAd-WT (i.n.)	10^8^ HAd-HA-NP (i.m.)	160	≤1.5
10^7^ HAd-WT (i.n.)	10^8^ HAd-HA-NP (i.n.)	120	≤1.5
10^8^ HAd-WT (i.n.)	10^8^ HAd-HA-NP (i.m.)	80	≤1.5
10^8^ HAd-WT (i.n.)	10^8^ HAd-HA-NP (i.n.)	22	2.3±1
10^8^ HAd-WT (i.n.)	5×10^8^ HAd-HA-NP (i.m.)	105	≤1.5
10^8^ HAd-WT (i.n.)	5×10^8^ HAd-HA-NP (i.n.)	91	≤1.5
10^9^ HAd-WT (i.n.)	10^8^ HAd-HA-NP (i.m.)	34	2.7±0.5
10^9^ HAd-WT (i.n.)	10^8^ HAd-HA-NP (i.n.)	22	2.3±0.2
10^9^ HAd-WT (i.n.)	5×10^8^ HAd-HA-NP (i.m.)	80	≤1.5
10^9^ HAd-WT (i.n.)	5×10^8^ HAd-HA-NP (i.n.)	52	≤1.5
PBS (i.n.)	10^8^ HAd-HA-NP (i.m.)	320	≤1.5
PBS (i.n.)	10^8^ HAd-HA-NP (i.n.)	183	≤1.5
PBS (i.n.)	5×10^8^ HAd-HA-NP (i.m.)	485	≤1.5
PBS (i.n.)	5×10^8^ HAd-HA-NP (i.n.)	278	≤1.5
10^9^ HAd-WT (i.m.)	5×10^8^ HAd-ΔE1E3 (i.n.)	10	6.5±0.1
10^7^ HAd-WT (i.m.)	10^8^ HAd-HA-NP (i.m.)	105	≤1.5
10^7^ HAd-WT (i.m.)	10^8^ HAd-HA-NP (i.n.)	91	≤1.5
10^8^ HAd-WT (i.m.)	10^8^ HAd-HA-NP (i.m.)	22	4.3±0.1
10^8^ HAd-WT (i.m.)	10^8^ HAd-HA-NP (i.n.)	60	≤1.5
10^8^ HAd-WT (i.m.)	5×10^8^ HAd-HA-NP (i.m.)	45	1.7±0.4
10^8^ HAd-WT (i.m.)	5×10^8^ HAd-HA-NP (i.n.)	69	≤1.5
10^9^ HAd-WT (i.m.)	10^8^ HAd-HA-NP (i.m.)	30	4.4±0.1
10^9^ HAd-WT (i.m.)	10^8^ HAd-HA-NP (i.n.)	10	4.0±0.1
10^9^ HAd-WT (i.m.)	5×10^8^ HAd-HA-NP (i.m.)	30	2.8±0.5
10^9^ HAd-WT (i.m.)	5×10^8^ HAd-HA-NP (i.n.)	34	3.2±0.1

Mice (10 animals/group) were inoculated i.m. or i.n. either with PBS (unprimed group) or with 10^7^, 10^8^, or 10^9^ p.f.u. of WT HAd5 (HAd-primed). Subsequently, naïve and HAd-primed mice were immunized i.m. or i.n. twice at four week interval with HAd-HA-NP. HAd-primed mice inoculated with HAd-ΔE1E3 (vector control) served as negative controls. Serum samples were obtained from all animals four weeks after the last immunization and analyzed by HI assay using a H5N1 reassortant virus and horse red blood cells. The titers are shown as geometric mean values (GM). Four weeks after the last immunization, mice from each group were challenged with 100-fold of 50% mouse infectious dose (MID_50_) of a H5N1 reassortant virus having HA and NA genes of A/Vietnam/1203/04 influenza virus. Three days post challenge mice were euthanized, and the lungs were collected. The lung viral titers were determined to evaluate the protective efficacy of the vaccine. The detection limit of the lung viral titer was ≥1.5 Log_10_ EID_50_/ml. HAd, human adenovirus; WT, wild type; i.m., intramuscular; i.n., intranasal.

In the 10^9^ i.n. HAd-primed group, i.n. immunization with HAd-HA-NP induced lower serum HI titers (22). Alternating with the i.m. route of immunization resulted in slight improvement in the HI titers (34). Increasing the vaccine dose by five-fold resulted in further improvement in HI titers in mice immunized either i.n. (52) or i.m. (80) indicating that the i.n.-induced (which mimics the natural route of infection in humans) vector immunity can be partially overcome by increasing the vaccine dose (P≤0.05). However, in the 10^9^ i.m. HAd-primed groups immunized with HAd-HA-NP, a HI titer of 30 was induced, and there were no significant changes in the titers by either alternating the route of vaccine inoculation or with an increased vaccine dose. These results indicate that the levels of vector immunity induced by i.m. priming with 10^9^ p.f.u. of HAd5-WT negatively impact the development of a humoral immune response against a HAd vector-based vaccine.

### Induction of cellular immune response in HAd-primed mice immunized with HAd-HA-NP

**Figure 3:**
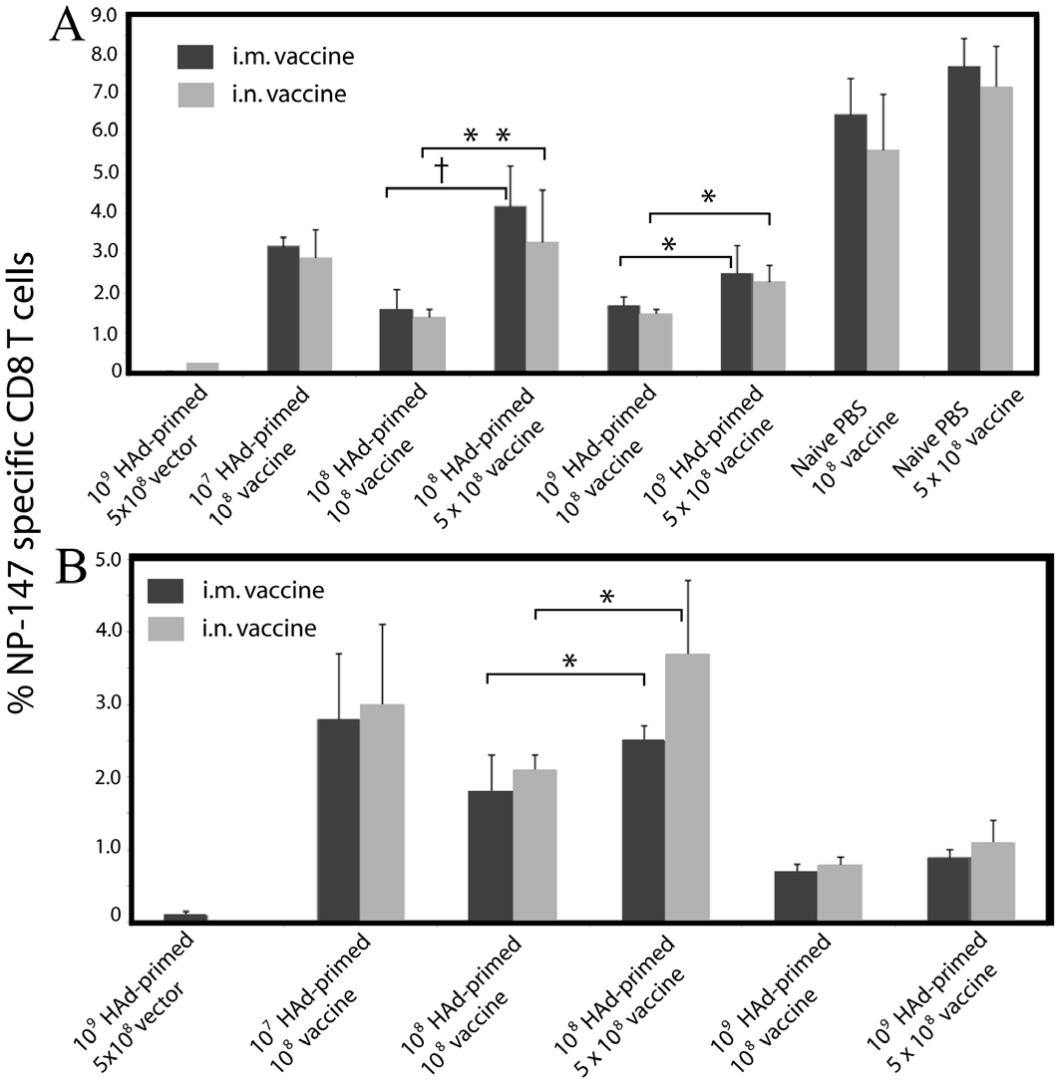
NP-147 epitope-specific CD8+ T cells in naïve or HAd5-primed mice immunized with HAd-HA-NP. Naïve or HAd-primed mice were immunized as described in the Materials and Methods. At four weeks after final immunization, animals were euthanized, and the spleens were collected. Single cell suspensions were prepared by passage through screens, and 1×10^6^ cells were stained with a murine MHC-encoded allele k^d^–specific pentamer for immunodominant NP-147 epitope-conjugated with phycoerythrin (PE) and also with anti-CD8 antibody-conjugated with allophycocyanin (APC) and anti-CD19 antibody-conjugated with flouro-isothiocyanin (FITC). Flow cytometeric analysis was done to identify the number of NP-147-specific CD8+ T cells. Data were collected using BD FACSCanto II (*BD Bioscience*, CA) and FACSDiva software was used for analysis. Data from five mice/groups are shown. (A) Percentage of NP-147-specific CD8+ T cells in intranasally primed groups. (B) Percentage of NP-147-specific CD8 T cells in intramuscularly primed groups. The error bars represent Mean ± SD from five animals/group: *, *P*≤0.05; **, *P*≤0.01; †, *P*≤0.001.

Cell-mediated immunity (CMI) plays an important role in virus clearance and thus contributes to the recovery from an influenza infection [22], [23]. As anticipated, the HAd-HA-NP vaccine elicited significantly higher percentages of NP-147 epitope-specific CD8 T cells in the naïve groups compared to the vector (HAd-ΔE1E3) control group (Figure 3A & 3B). The percentages of NP-147 epitope-specific CD8 T cells in 10^7^ HAd-primed groups (i.n. or i.m.) was significantly higher than in vector control groups following immunization with HAd-HA-NP by either the i.n. or i.m. route. However, the percentages NP-147-specific CD8 T cells were 1.5–2 fold lower in HAd-primed groups (i.n. or i.m.) compared to naïve groups, suggesting that preexisting vector immunity had a modest effect on the induction of CMI following immunization with HAd-HA-NP (Figure 3A & 3B).

The percentages of NP-147 epitope-specific CD8 T cells in 10^8^ HAd-primed groups were two-fold lower compared to the 10^7^ HAd-primed groups (i.n. or i.m.) following immunization with HAd-HA-NP by either route (Figure 3A & 3B). Interestingly, increasing the vaccine dose by five-fold resulted in significantly higher percentages of NP-147 epitope-specific CD8 T cells in the 10^8^ HAd-primed groups compared to both 10^7^ and 10^8^ HAd-primed groups receiving the lower dose (10^8^ p.f.u.) of the vaccine. As expected, a further increase in the level of preexisting vector immunity led to a further decrease in the percentages of NP-147 epitope-specific CD8 T cells. Furthermore, an increase in the vaccine dose by five-fold resulted in significantly higher percentages of NP-147 epitope-specific CD8 T cells in the 10^9^ i.n. primed group compared to the 10^9^ i.n. primed group receiving the lower vaccine dose (10^8^ p.f.u.). However, this increase was not noticeable in the 10^9^ i.m. primed group.

**Figure 4:**
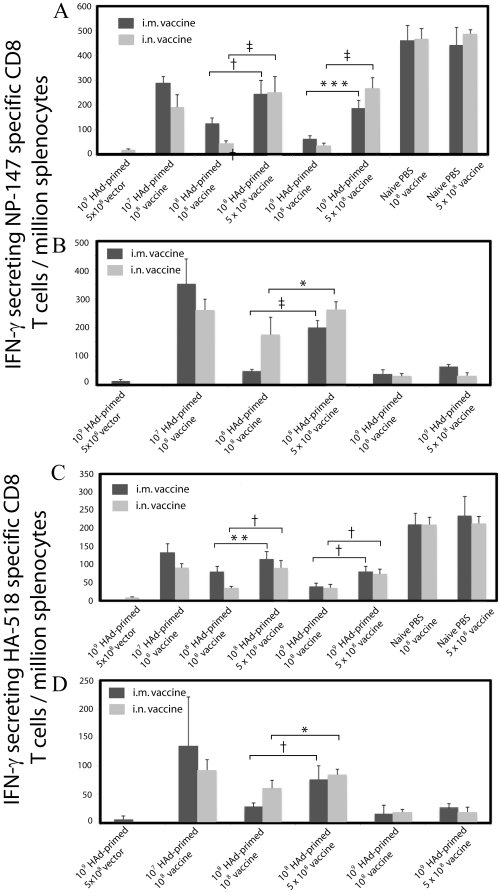
ELISpot measurements of IFN-γ expression in spleen cells of naïve or HAd5-primed mice immunized with HAd-HA-NP. Naïve or HAd5-primed mice were immunized as described in the Materials and Methods. At four weeks after final immunization, animals were euthanized, and the spleens were collected. Single cell suspensions were prepared by passage through screens, and 1×10^6^ cells were cultured in the presence of HA-518 or NP-147 peptide on anti-interferon-γ antibody-coated 96-well filter plates and developed according to an ELISpot protocol. Splenocytes cultured in presence of phorbol myristate acetate (PMA) and ionomycin served as positive controls in each group. The ELISpot plates were read using a Bioreader 5000 (*BIOSYS*, Miami, FL). (A) Number of IFN-γ-secreting NP-147-specific CD8 T cells in intranasally (i.n.) primed groups. (B) Number of IFN-γ-secreting NP-147-specific CD8 T cells in intramuscularly (i.m.) primed groups. (C) Number of IFN-γ-secreting HA-518-specific CD8 T cells in i.n. primed groups. (D) Number of IFN-γ-secreting HA-518-specific CD8 T cells in i.m. primed groups. The error bars represent Mean ± SD from five animals/group: *, *P*≤0.05; **, *P*≤0.01; ***, *P*≤0.005; †, *P*≤0.001; ‡, *P*≤0.0001.

The functionality of HA-518 and NP-147-specific CD8 T cells was assessed by enumerating interferon-γ (IFN-γ) expressing cells by ELISpot assay. Significantly higher numbers of IFN-γ-secreting HA-518- and NP-147-specific CD8 T cells were detected in the spleens from the naïve groups immunized i.n. or i.m. with HAd-HA-NP compared to the vector control groups following stimulation with the HA-518 (*P*≤0.0001) or NP-147 (*P*≤0.0001) peptide, respectively (Figure 4A–D). In general, the number of IFN-γ-secreting HA-518- or NP-147-specific CD8 T cells in the spleens of HAd-primed groups immunized with the vaccine were lower compared to the naïve immunized groups, and a five-fold increase in the vaccine dose resulted in an increase in the number of IFN-γ secreting HA-518- or NP-147-specific CD8 T cells in all the primed groups except for the 10^9^ i.m. primed groups (Figure 4A–D). Overall, it seems that with the increase in preexisting HAd5-neutralizing antibodies, there was a titer-dependent decline in the CMI response which significantly improved with an increase in the vaccine dose by five-fold. There was excellent correlation between the ELISpot and NP pentamer staining data.

### Protection of HAd-primed mice immunized with HAd-HA-NP following challenge with a reassortant H5N1 influenza virus

Unprimed mice immunized with HAd-HA-NP had lung viral titers on day 3 post-challenge below the level of detection (1.5 Log_10_ EID_50_/ml). Similarly, the 10^7^ HAd-primed groups (i.n. or i.m.) immunized with HAd-HA-NP had lung viral titers at or below the level of detection (1.5 Log_10_ EID_50_/ml) indicating that the preexisting vector immunity did not adversely impact the protective efficacy. However, the 10^8^ HAd-primed groups (i.n. or i.m.) immunized with HAd-HA-NP by the same route that was used for priming exhibited less efficient virus clearance from the lungs. Interestingly, either using a different route of inoculation for priming and vaccination or increasing the vaccine dose by five-fold resulted in lung viral titers at or below the level of detection. In the 10^9^ i.n. HAd-primed groups (i.n. or i.m.) immunized with HAd-HA-NP by the same route that was used for priming, there was only partial (approximately 2–4 logs) virus clearance. By changing the route of priming and immunization, complete protection was observed only in the group where the i.n. priming was followed by a five-fold increase in the vaccine dose administered by either route (i.m. or i.n.). Even a five-fold increase in the vaccine dose did not yield complete protection in the 10^9^ i.m. HAd-primed groups immunized with HAd-HA-NP by either route (i.n. or i.m.) suggesting that the level of preexisting vector-neutralizing antibody titer could serve as an indicator for predicting the efficacy of Ad-based vaccines.

## Discussion

To meet the global vaccine demand in a pandemic, various egg-independent vaccine strategies need to be explored to supplement egg-dependent influenza vaccine approaches. Ad vector-based influenza vaccines have been shown in clinical studies to be safe and immunogenic in humans [24], [25]. The strong innate and adaptive immune responses induced by Ad vectors impart adjuvant-like properties facilitating better immune responses against the transgene product/s. However, several preclinical studies have suggested that the presence of preexisting HAd-specific neutralizing antibodies might inhibit the generation of immune responses against the expressed immunogen [1], [14], [18]. In the present study, we evaluated the role of preexisting HAd5-specific neutralizing antibodies in inhibiting the immunogenicity and efficacy of a HAd5 vector-based H5N1 influenza vaccine in a mouse model. Low levels of vector immunity (<520 virus-neutralization titer) did not seem to adversely affect the protective vaccine efficacy, while further increases in vector immunity were taken care of by using an alternate route of immunization or by an increase in the vaccine dose. The importance of NP-specific CD4 T cells and non-neutralizing antibodies in the virus clearance was not pursued in this study.

To mimic the natural exposure of HAd to the majority of humans, we primed mice with HAd5 by the i.n. route to establish the state of preexisting vector immunity since many HAd infect via the mucosal route. The i.m. HAd5-primed groups represented the development of HAd-specific immune responses following i.m. immunization with a HAd vector-based vaccine. We attempted to circumvent the inhibitory effect of high levels of preexisting vector immunity by either a change in the route of vaccine inoculation or an increase in the vaccine dose.

In the presence of preexisting neutralizing antibodies (300–520), there was a modest decline in the levels of CMI and HI levels in response to immunization with HAd-HA-NP. This level of immune response was sufficient enough to provide excellent protection against the challenge with a H5N1 reassortant virus. There were lower levels of humoral and cellular immune responses after the i.n. immunization compared to the i.m. immunization which is consistent with earlier findings [11], [26]–[28].

Further increase in the levels of vector-specific neutralizing antibody response (1000–1480) resulted in a greater decline in influenza virus-specific immune responses with the inhibition more pronounced when the route of inoculation for Ad-priming and influenza virus immunization were the same (e.g., i.n. and i.n., i.m. and i.m.). This level of vector immunity could be overcome either by changing the route of priming and immunization or by increasing the vaccine dose by five-fold. To test the upper limit of vector immunity that could be tolerated without adversely affecting the vaccine protective efficacy, the vector-specific neutralizing antibody titer was raised to 2240 by i.n. priming with high doses of HAd5. The inhibitory effect was partially overcome by the five-fold increase in the vaccine dose, and the resultant immune response was sufficient to provide complete protection. Further increase in the level of vector-specific neutralizing antibody titer to 3040 by i.m. priming with HAd5 only provided partial protection even in the groups receiving a high vaccine dose.

Alternating the route of priming and immunization was partially successful in overcoming vector immunity thus indicating the potential role of the route of inoculation in developing the level of humoral and CMI responses. It has been suggested that the route of vaccination impacts the magnitude, phenotype and trafficking of antigen-specific CD8 T cells in mice [29], [30]. A HAd-based HIV vaccine also showed some inhibition in eliciting immunogen-specific immune responses in the presence of vector immunity, however, this effect was minimized by increasing the vaccine dose [31]. Nevertheless, in a clinical trial with a HAd-based influenza vaccine, there were no strong correlations between vector immunity levels and a decrease in vaccine efficacy [25]. The inhibitory effect of vector immunity was more pronounced for the humoral immune response compared to the CMI response which is consistent with previous reports [32]–[34]. Taken together, our data clearly show that the magnitude of humoral and cellular immune responses to Ad-vectored vaccine antigens depend upon the levels of preexisting antibodies against the vector. However, vector-specific immune responses can be overcome by increasing the antigen dose or by administering antigen by a different route. In conclusion, based on the present study alone, it will be difficult to predict the range of the vector immunity in humans that can be tolerated or overcome by either increasing the dose or changing the route of administration of the HAd5 vector-based vaccines. Nevertheless, the study does highlight the importance of exploring these strategies in humans to improve the outcome of HAd5 vector-based vaccines.

## Materials and Methods

### Ethics Statement

The Purdue University Biosafety Committee and Animal Care and Use Committee approved the protocol for all animal studies at Purdue University, under the auspices of the Institutional Animal Care and Use Committee (IACUC) #A3231-01 which is supported by the American Association for Laboratory Animal Science (AALAS). The 293 cell line was obtained commercially from American Type Culture Collection (ATCC), and the 293Cre cell line was obtained from Merck & Co. The BHH2C cell line which was created in the PI's laboratory used a combination of commercially available cell lines, MDBK and 293. The use of all human cell lines and the construction of BHH2C hybrid cell line were with permission from the Purdue University Institutional Review Boards (IRB) formed in accordance with federal regulations. A Research Exemption was obtained for the use of commercially available human cell lines. The IRBs are a unit of the Human Research Protection Program (HRPP) which is housed within the Office of Research Administration (ORA).

### Cell lines and recombinant viruses

293 (human embryonic kidney cells expressing HAd5 E1 gene products; obtained from ATCC) and 293Cre (293 cells that constitutively expresses *Cre*-recombinase enzyme (a gift from *Merck Inc.*, Whitehouse Station, NJ) [35] were grown as monolayer cultures in Eagle's minimum essential medium (MEM) (*Life Technologies*, Gaithersburg, MD) and supplemented with 10% reconstituted bovine serum (Fetal Clone III; *Hyclone*, Logan, VT) and 50 µg/ml gentamycin. All constructs were purified by cesium chloride density-gradient centrifugation and titrated by plaque assays on a hybrid cell line using MDBK and 293 cell lines (BHH2C) [36] as described previously [37]. The construction and propagation of replication defective HAd-ΔE1E3 (HAd5 vector having deletions in the E1 and E3 regions) has been previously described [11]. HAd5-WT virus was purified by cesium chloride density-gradient centrifugation and titrated by plaque assay on BHH2C cells.

### Generation of replication deficient HAd-HA-NP vector

A *Cre*-recombinase-mediated site-specific recombination technique [20] was used to insert the full-length coding region of the HA gene (with a modified polybasic site) of the A/Vietnam/1203/04 (H5N1) influenza virus under the control of the CMV promoter and BGH polyA. The polybasic cleavage site QRERRRKKR↓G present in the HA gene of A/Vietnam/1203/04 (H5N1) influenza virus was modified to QRETR↓G to reduce the rare possibility of genetic exchange between the HA in HAd-HA-NP vaccine and a circulating strains of influenza A virus. The full-length coding region of NP gene of the A/Vietnam/1203/04 (H5N1) virus under control of the murine CMV promoter and the SV40 polyA was similarly inserted into the E1 region of the HAd genome. Both genes in HAd-HA-NP were in the E1-parallel orientation. The recombinant virus was plaque purified, and its genome was analyzed by restriction enzyme digestions to confirm the presence of HA and NP gene cassettes and the absence of any other major deletion or insertion.

### Western blot analysis

293 cells were mock-infected or infected with an empty vector (HAd-ΔE1E3) or HAd-HA-NP at a multiplicity of infection (m.o.i.) of 20 p.f.u. per cell. Cells were harvested 36 h post-infection, and cell lysates were prepared and analyzed by Western blot [38] with a polyclonal serum against H5 HA (obtained from Immunology and Pathogenesis Branch, Influenza Division, Centers for Disease Control and Prevention, Atlanta, GA, USA) and a monoclonal antibody against NP (obtained from Immunology and Pathogenesis Branch, Influenza Division, Centers for Disease Control and Prevention, Atlanta, GA, USA) to confirm the expression of HA and NP proteins. Mock (PBS) or HAd-ΔE1E3 served as negative controls.

### Induction of vector-specific immunity and evaluation of vaccine efficacy

6–8 week old female BALB/c mice (10 animals/group) were inoculated by either the i.m. or i.n. route with a single dose of 10^7^, 10^8^, or 10^9^ p.f.u. of HAd-WT. These groups were referred to as HAd-primed groups. The unprimed (naïve) mice were similarly inoculated with phosphate-buffered saline (PBS). Four weeks after priming, mice were bled by retro-orbital puncture to evaluate the development of HAd-specific neutralizing antibody titers. HAd-primed and naïve (PBS-inoculated) mice were subsequently immunized twice (4 weeks apart) with 1×10^8^ or 5×10^8^ p.f.u. of HAd-HA-NP vaccine by either the i.m. or i.n. route. Additional groups of HAd-primed mice were similarly immunized with 5×10^8^ p.f.u. of HAd-ΔE1E3 (vector control) to serve as negative controls.

Four weeks after final immunization, blood samples were collected through retro-orbital puncture to evaluate the development of HA-specific antibodies. Five animals from each group were euthanized to collect the spleen cells to evaluate the induction of HA- and NP-specific CMI responses. The remaining mice from each group were challenged with 100-fold of 50% mouse infectious dose (MID_50_) of a reverse genetics derived A/Puerto Rico/8/1934(H1N1) [PR8] containing HA and NA gene fragments of A/Vietnam/1203/04 (H5N1) [VNH5N1-PR8/CDC-RG] [24]. Since this reassortant virus is not lethal and does not produce clinical disease and weight loss in mice, protection efficacy was monitored by viral clearance in the lungs. Three days post-challenge, mice were euthanized, and the lungs were collected to determine viral titers to evaluate protective efficacy. Briefly, thawed lung tissues were homogenized in 1 ml of sterile PBS. These lung homogenates were then titrated in 10-day-old embryonated eggs in a 10-fold dilution, and positive eggs were identified by hemagglutination of horse red blood cells with allantoic fluid. Values were expressed as log10 EID50/ml ± SEM. The limit of virus detection was set as 1.5 log10 EID50/ml [39]. The NP genes are fairly conserved between A/Vietnam/1203-04 (in the Ad vector) and A/PR8/34 (the backbone of the challenge virus); there is 93% identity at the amino acid level and 85% identity at the nucleotide level. One of the major CD8 T cell epitopes (NP147) is 100% conserved in between A/Vietnam/1203-04 and A/PR8/34.

### Serological assays

HAd neutralizing serum antibody titers were determined as previously described [13]. The virus neutralization titer was the reciprocal of the highest serum dilution that completely prevented the development of c.p.e. Pre-challenge serum samples were analyzed for the presence of HI antibody titers using horse red blood cells as described previously [40].

### NP-specific pentamer staining

Splenocytes were isolated and stained with a murine MHC k^d^–specific pentamer for immunodominant NP-147 epitope (*Proimmune Inc.*, Bradenton, FL) conjugated with phycoerythrin (PE) and an anti-CD8 antibody conjugated with Allophycocyanin (APC) (*BD PharMingen*, San Jose, CA.) as described previously [11]. B cells were removed by staining splenocytes with anti-CD19 flouro-isothiocyanin (FITC) and gating them out in analysis. Flow cytometric analyses were done using BD FACSCantoII, (*BD Bioscience*, San Jose, CA) to identify the percent NP-147 epitope-specific CD8+ T cells among the total splenic CD8+ T cells.

### ELISpot assay

96-well filter plates (*Millipore*, Bedford, MA) were coated with an anti-mouse interferon gamma (IFN-γ) antibody (*BD Bioscience*, San Jose, CA) and incubated at 4°C overnight. Splenocytes (3.3×10^5^ to 1×10^6^ cells per well) from each mice were cultured in the presence of HA-518 or NP-147 peptides in RPMI medium (*GIBCO*, Grand Island, NY), supplemented with 10% reconstituted fetal bovine serum for 60 h and developed according to an ELISpot protocol [41]. Splenocytes cultured in the presence of phorbol myristate acetate (PMA) and ionomycin (*Sigma-Aldrich, Inc*., St. Louis, MO) served as positive control within each group.

### Statistical analysis

Log-transformation of titer measurement was assessed by Shapiro-Wilktest, found to be normally distributed and used for the analysis using SAS 9.2. Tukey's multiple comparison was used for calculation of significance. The significance was set at *P* <0.05.
